# Clinical Assessment of Acute Organophosphorus Pesticide Poisoning in Pediatric Patients Admitted to the Toxicology Emergency Department

**DOI:** 10.3390/toxics10100582

**Published:** 2022-10-02

**Authors:** Ioana-Cezara Caba, Violeta Ștreangă, Mona-Elisabeta Dobrin, Cristina Jităreanu, Alexandra Jităreanu, Bianca-Ștefania Profire, Maria Apotrosoaei, Alin-Viorel Focșa, Bogdan Caba, Luminița Agoroaei

**Affiliations:** 1 Department of Toxicology, Faculty of Pharmacy, University of Medicine and Pharmacy “Grigore T. Popa”, 700115 Iasi, Romania; 2 Department of Toxicology, “Saint Mary” Children Emergency Hospital, 700309 Iasi, Romania; 3 Department of Clinical Biochemistry, Clinical Hospital of Pulmonary Disease, 700115 Iasi, Romania; 4 Department of Internal Medicine, Faculty of Medicine, University of Medicine and Pharmacy “Grigore T. Popa”, 700115 Iasi, Romania; 5 Department of Pharmaceutical Chemistry, Faculty of Pharmacy, University of Medicine and Pharmacy “Grigore T. Popa”, 700115 Iasi, Romania; 6 Department of Drug Industry and Pharmaceutical Biotechnology, Faculty of Pharmacy, University of Medicine and Pharmacy “Grigore T. Popa”, 700115 Iasi, Romania; 7 Department of Biomedical Sciences, Faculty of Medical Bioengineering, University of Medicine and Pharmacy “Grigore T. Popa”, 700115 Iasi, Romania

**Keywords:** organophosphate pesticide poisoning, toxicity of pesticides, pediatric patients, biochemical parameters, clinical toxicology assessment

## Abstract

Pesticide poisoning in pediatric patients is still an important reason for presenting to the emergency department in Romania. In this context, the present study aims to raise awareness of the toxicological impact of pesticides on human health in pediatrics. For this purpose, the demographic characteristics, clinical assessment, and outcome of pediatric patients with acute pesticide poisoning admitted to the toxicology department of “Saint Mary” Emergency Children’s Hospital from Iasi, were analyzed. This retrospective study focused on the clinical and laboratory data of patients aged under 18 years diagnosed with acute pesticide poisoning between 2010–2020. The statistical analysis was performed using the Statistica 10 package. A total of 49 patients presented with manifestations of acute pesticide poisoning, and the most common pesticide involved was diazinon. The most frequent exposure route was accidentally ingesting pesticide products (95%). The primary clinical manifestations were toxic encephalopathy, coma, depressive disorder, gastric disorders, and respiratory failure. Changes in the glycemic status, liver, and kidney damage were also present. Treatment included decontamination, administration of antidote, supportive care, and recommendations to be closely monitored to avoid a new incident. These results highlight the toxic potential of pesticides on human health and their biological consequences, which require an increase in consciousness of the precautions imposed on their use, especially when children are nearby.

## 1. Introduction

A global public health concern nowadays consists of poisoning from pesticides. Every year it accounts for nearly 300,000 deaths worldwide. The danger of pesticide exposure is inevitable; humans are exposed in multiple ways to these compounds. An important factor is the mode of exposure; it also signifies the concentration of pesticide exposure and the impact on human health [1]. The principal route of exposure to pesticides for the general population is ingesting possible contaminated food products with residues of insecticides, herbicides, or fungicides sprayed on the crops. Pesticide-based products are used not only in agriculture but also in public and domestic applications that provide additional routes of exposure for citizens, including children, mainly through inhalation and direct skin contact. Moreover, exposure can affect workers, primarily pesticide handlers [2].

The need to assess the health effects arising from their use in various activities is increasingly recognized. People and animals are exposed daily to complex chemical mixtures, such as pesticides [3]. Many pesticides can produce acute and chronic side effects when individuals are exposed, such as damage to the nervous system (central or peripheral), the neuroendocrine system, altered metabolic changes, reproductive functions, or even mutations and cancer [1,4]. Epidemiological studies have shown some harmful effects of pesticides on various organs, including the liver, brain, lungs, and colon, and some recent investigations have demonstrated fatal consequences, such as cancer [1,5]. An association between acute exposure to pesticides and the subsequent development of seizures was noticed in the literature, but, still, no study has shown a specific relationship between long-term exposure to low doses of pesticides and epilepsy. In their research, Requena et al. described a higher prevalence of diagnosed epilepsy in areas where pesticides have been used in intensive agriculture under plastic greenhouses [6]. Acute childhood leukemia, neurodevelopmental effects, cardiotoxicity, or respiratory failure are adverse effects of pesticide exposure. These rising interest of researchers in these subjects remains inconclusive and inherently limited by heterogeneous exposure assessment and statistical studies [7,8,9,10].

The study of the specialized literature shows that exposure to pesticides has several clinical and biochemical implications, through lower levels of serum acetylcholinesterase and significant changes in some laboratory parameters. Exposure to organophosphorus pesticides is primarily evaluated by the level of AChE, which was observed to be significantly lower among exposed individuals [11]. Biochemical examination following exposure to pesticides was associated with liver damage, through a significant increase in ALT (Alanine aminotransferase), AST (Aspartate aminotransferase), GGT (Gamma-glutamyl Transferase), as well as high levels of ALP (Alkaline phosphatase) [12]. Hematological changes in pesticide-exposed subjects showed a decrease in mean corpuscular hemoglobin (MCH), a significant decrease in hematocrit and hemoglobin, and an increase in the number of white blood cells (WBC), and platelets (PLT) [13]. In addition, hyperglycemic changes during poisoning with organophosphorus pesticides were also mentioned in the literature, and the glycemic state was correlated with the severity of toxicity, the level of serum cholinesterase, and the dose of atropine required for complete recovery [14].

The potential toxicity of pesticides is a public health problem because it applies almost anywhere, on agricultural land, roads, and in residential, educational, and leisure facilities. A genuine concern is acute organophosphorus pesticide poisoning, especially in rural areas, and the intentional ingestion of these compounds because it frequently leads to patient death [15]. Suwannakul et al. describe that organophosphate pesticide exposure in early and late pregnancy can influence different aspects of infant developmental performance [16]. However, there is a lack of data regarding the exposure of the general human population to these products [17,18]. In addition, pesticides in tap water, food, honey, and natural products used as medicines, represent a growing concern for producers and consumers [19,20,21].

A critical category exposed to pesticides in different ways is pediatric patients. Young children can be exposed to pesticides with possible effects on their health, both in childcare centers and in their family homes [22]. Significant cases of accidental or voluntary acute poisoning with these compounds are described in the scientific literature [23,24,25,26,27,28]. In addition to acute poisoning, chronic exposure to low-level pesticides can significantly increase threats to long-term health [22]. Children in critical stages of development are particularly vulnerable. There are correlations mentioned in studies between pesticide exposure and degradation of psychomotor and mental development, attention deficits, and childhood cancer (leukemia and brain tumors). Children’s hand-to-mouth activity and playing close to the ground make them more susceptible to chemical exposure from surfaces and internal dust. The risks of acute and chronic poisoning in children are higher because the capacity for toxic chemical metabolization is lower, and the exposure is higher due to a greater intake of food, water, and air per body weight than in adults [22]. From another point of view, the colorful appearance of some home pesticides and their easy-access packaging can increase the danger to young children [24].

Many children from rural areas in Romania are exposed to this danger due to the intensive use of pesticides by families in agricultural activities. The accidental ingestion of pesticides can occur since children are sometimes not under the strict supervision of their parents or relatives. Children should not have access to chemicals that are so dangerous for their health. Reports on accidental poisonings in children from northeast Romania over certain periods have been described before [29]. However, to our knowledge, no study has evaluated the correlations between clinical or biochemical parameters with the toxicological potential of organophosphorus pesticides. There is also a lack of data regarding the characteristics of pediatric organophosphate poisoning in our region. For this purpose, this study aims to evaluate the variability of clinical and biochemical parameters in acute organophosphorus pesticide poisoning in pediatric patients and the changes in laboratory parameters depending on the diagnosis at admission. This kind of information can be useful for a rapid diagnosis and treatment of pesticide poisoning, which sometimes is crucial for the lives of the patients [28].

## 2. Materials and Methods

### 2.1. Study Group

This study is a retrospective clinical assessment. The patients selected for this study were aged between 1 month and 18 years, presenting with confirmed acute organophosphorus pesticide poisoning, in the Regional Center of Toxicology from the most prominent Emergency Pediatric Hospital in the North-Eastern part of Romania, “Saint Mary” Hospital, in the period 2010–2020. Data were collected from the electronic medical records of pediatric patients. This retrospective study focuses on statistical reports and analyzed the demographic and laboratory data of 49 pediatric patients diagnosed with acute intoxication with different organophosphorus pesticides during a period of ten years. There were no other extra inclusion criteria for the patients in the study, only the age and the diagnosis of acute organophosphate pesticide poisoning during the study period. The information from the anamnesis and the clinical and biological parameters contributed to identifying the compound responsible for the poisoning. The study excluded subjects who had missing laboratory data.

### 2.2. Determination of Laboratory Parameters

The hematological and biochemical parameters were determined at the time of admission to the hospital, with acute organophosphorus poisoning diagnosis, in the hospital laboratory, using the available equipment.

#### 2.2.1. Hematological Assay

White blood cells (WBC), red blood cells (RBC), hemoglobin (HGB), hematocrit (HCT), platelet count (PLT), platelet distribution width (PDW), platelet large cell ratio (P-LCR), plateletcrit (PCT), mean corpuscular volume (MCV), mean corpuscular hemoglobin (MCH), mean corpuscular hemoglobin concentration (MCHC), mean platelet volume (MPV), RBC distribution width-standard deviation (RDW-SD), RBC distribution width-coefficient of variation (RDW-CV), neutrophil percent (N%), lymphocyte percent (L%), monocyte percent (M%), eosinophil percent (E%), basophil percent (B%) were assessed using Hematology Analyzers Mindray, Sysmex_XN1000.1, and Pentra 60.

#### 2.2.2. Biochemical Assay

Serum parameters were measured using biochemical auto-analyzers BA 400, Laburised, and Randox Rx Imola. All tests were performed at the Biochemical Laboratory of “Saint Mary” Emergency Children Hospital, Iasi, following standard procedures for clinical biochemistry purposes. The biological markers measured were alanine aminotransferase (ALT), glucose, aspartate aminotransferase (AST), cholinesterases (ChE), urea, and creatinine.

### 2.3. Statistical Analysis

All statistical results were obtained using the Statistica 10 package (StatSoft Inc., Tulsa, OK, USA). Descriptive statistic was performed using the Microsoft Excel program and for each biochemical and hematological parameter determined in patient’s average, standard deviation, median, and range. The normality of the results was tested using the Kolmogorov–Smirnov test. The Spearman correlation coefficient was determined to measure the correlations between clinical parameters of all investigated subjects. A *p*-value of <0.05 was considered statistically significant. Discriminant function analysis and the Mann–Whitney U test were used to determine whether age groups of pediatric patients exposed to organophosphorus pesticides differed in investigated clinical parameters. To determine the elements of the highest significance in the model, the following parameters were used: partial Wilks’ lambda, the F-test of partial Wilks’ lambda, and its *p*-value.

### 2.4. Ethics Considerations

The Institutional Ethical Committee of “Saint Mary” Emergency Children Hospital, Iasi, a reference center in the Moldova region of Romania for diagnosing and treating pediatric diseases, approved the study (No. 26564/15 September 2021).

## 3. Results

### 3.1. Clinical and Demographic Characteristics of the Patient’s Groups

A total of 48 pediatric patients were exposed accidentally (*n* = 39) or voluntarily (*n* = 9) to toxic products based on organophosphorus pesticides used in pest removal. In addition, there was one death after the unintentional ingestion of organophosphate compound (diazinon). The most common conditions of hospitalized patients were: toxic encephalopathy, coma, depressive disorder, gastric disorders, and respiratory failure (Table 1).

Accidental exposure to organophosphorus pesticides occurred at home, and the route of contamination was primarily through ingestion (n = 46) and dermal absorption (n = 2). Most pediatric patients exposed to accidental intoxication with organophosphorus pesticides came from rural (n = 44) and urban (n = 4) areas and were 1–5 years old (n = 31). 

### 3.2. Profile of the Hematological Parameters

Complete blood count (Table 2) and some biochemical parameters (Table 3) were determined for each patient after acute exposure to organophosphorus pesticides. As seen in Table 2, children aged 1–5 years showed low values for RBC, HCT, and HGB, compared to the other assessed age ranges, 6–11 years and 12–17 years.

The significance of the statistical difference between the age groups evaluated in the pediatric patients investigated was analyzed using the non-parametric Mann–Whitney U test. Thus, the results showed statistically significant differences, especially between the groups aged 1–5 years and 12–17 years, differences given by the following biological parameters: HGB (*p* = 0.003), HCT (*p* = 0.007), MCV (*p* = 0.0001), MCH (*p* = 0.0001), RDW-cv (*p* = 0.01), RDEW-SD (*p* = 0.029), *p*-LCR (*p* = 0.020), PDW (*p* = 0.036) (See Table S1 in the Supplementary Materials).

Leukocytosis was present through the high number of leukocytes, especially in the group of children aged 1–5 years (mean = 16.83).

The results of the investigated patients showed low values of serum cholinesterase for the children aged 1–5 years, with values of average concentration = 1449.42 IU/L, but also for the group aged 12–17 years (average concentration = 1211 IU/L), values that are much lower than the biological reference range (5400–13,200 IU/L).

Statistical correlations between serum ChE levels and biological parameters were studied. The correlation matrix applied to the dataset highlighted correlations of CHE, especially with ALT, AST, PLT, creatinine, glucose, and RDW-cv (Table 4). The resulting correlations point in particular to an association of ChE values with impaired liver and kidney function and carbohydrate metabolism.

Descriptive statistics performed for biochemical parameters show high values of serum creatinine, especially for the group aged 1–5 years, some pediatric patients having values even of 1.02 mg/dL, values much higher than the biological reference range (0.25 mg/dL).

Laboratory results (Table 3) scored high values for ALT, with a mean concentration value ± stdev = 46.94 ± 123.58 IU/L) for the age range 1–5 years compared to the age range 6–11 years (mean concentration ± stdev = 21.67 ± 11.97 IU/L) and range 12–17 years (mean concentration = 13.27 ± 6.85). In the case of results for AST, the growth trend was in children with an age range of 1–5 years (mean concentration = 66 ± 161.69), with higher results than the other groups considered, 6–11 years (mean concentration = 30 ± 8.70), and 12–17 years (mean concentration = 13.27 ± 6.85).

**Figure 1:**
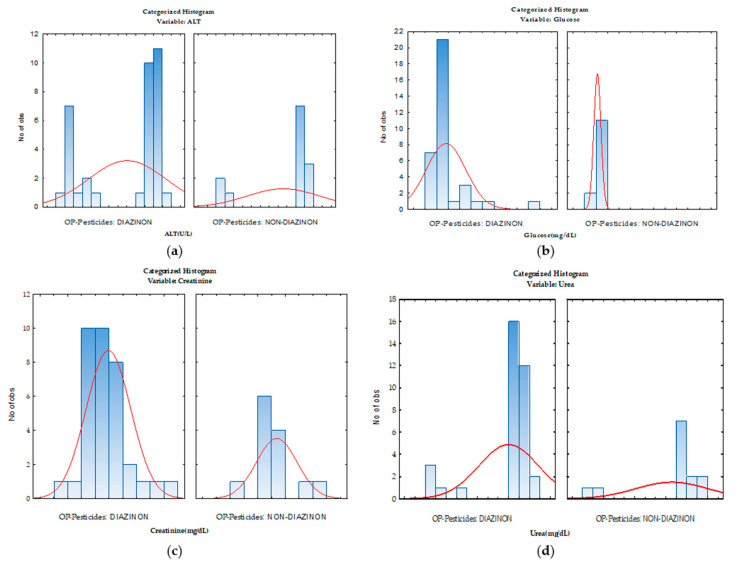
Distribution of ALT (**a**), glucose (**b**), creatinine (**c**), and urea (**d**) concentrations by incriminated substance (diazinon/non-diazinon compound).

Moreover, evaluating the results of this study according to the incriminated toxic substance (diazinon/non-diazinon), a different distribution of biochemical parameters was observed that assessed renal function (urea, creatinine), liver (ALT), and glycemic status for all pediatric patients (Figure 1).

Depending on the age of the subjects, the biochemical and hematological profile of the patients with acute organophosphorus pesticide intoxication was followed, with the help of the statistical processing program of the experimental data. Thus, in the default algorithm of the method data were introduced on 48 patients and 25 characteristics as well as three age ranges (1–5 years, 6–11 years, 12–17 years) of pediatric patients intoxicated with organophosphorus pesticides. The results of the discriminated analysis highlighted five clinical parameters that contributed significantly (*p* ˂ 0.05) to the separation of the classes considered. Table 2 presents the statistical results of selecting the essential variables for evaluating biochemical and hematological parameters according to the age groups considered for the subjects included in the study.

**Figure 2:**
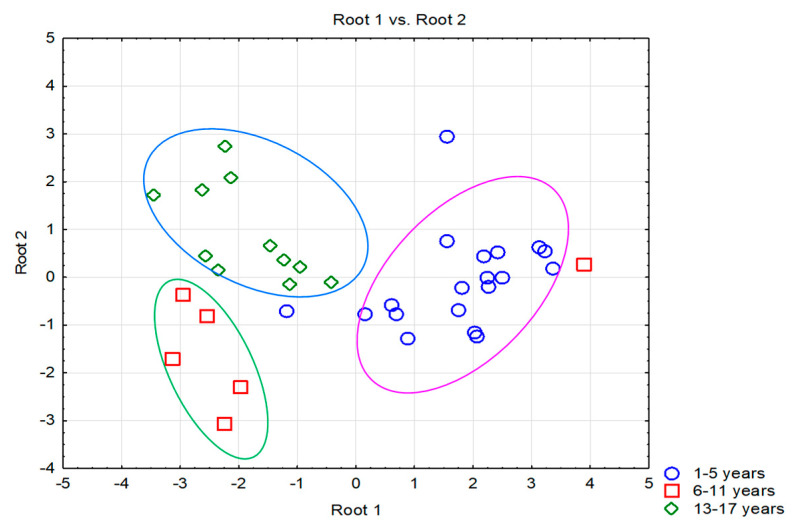
Graphical representation of scores corresponding to subjects with organophosphorus pesticide poisoning by age.

Thus, the statistical results point out that out of the 12 variables retained in the model, only five contributed significantly to the separation of classes (Table 5), respectively, the age ranges considered. Thus, based on the statistical results obtained, it can be concluded that the number of leukocytes (F = 9.55, *p* = 0.001), serum urea (F = 7.44, *p* = 0.004), ALT (F = 3.60; *p* = 0.047), serum creatinine (F = 4.67; *p* = 0.022), and the number of lymphocytes (F = 4.25; *p* = 0.029) are the biological parameters with the most significant importance in discrimination (See Table S2 in the Supplementary Materials). The scatterplot representing discriminant functions (Figure 2) shows an excellent separation between all age groups. At the same time, the Mann–Whitney U test shows a statistically significant difference (*p* ˂ 0.05) for ALT concentrations between the groups aged 1–5 years and 12–17 years.

**Figure 3:**
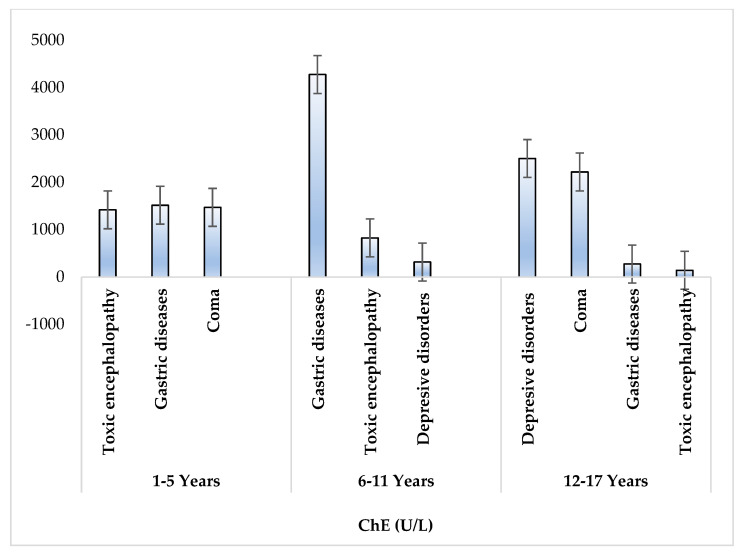
Mean serum ChE concentration (IU/L) levels in pediatric patients by age and diagnosis.

Toxic encephalopathy and gastric disorders were characteristic of all age groups investigated, while depressive disorder was characteristic of age groups 6–11 years and 12–17 years. Children diagnosed with toxic encephalopathy and depressive disorders recorded the lowest serum ChE values (Figure 3). At the same time, as stated at admission, nine of the investigated patients declared voluntary intoxication with organophosphorus pesticides, aged between 11–17 years.

It has been observed that if patients survive the first days of intoxication, their condition also presents with psychotic episodes, aggressive events, and personality changes due to exacerbations of pre-existing psychiatric problems. Comparing the results of the laboratory parameters of pediatric patients according to the age range and the diagnosis at admission, a statistically significant difference is observed in patients diagnosed with toxic encephalopathy and gastric conditions (*p* ˂ 0.05), for the number of eosinophils and the concentration of ChE.

## 4. Discussion

Accidental or voluntary pesticide poisonings in pediatric patients are highly prevalent in most countries, despite the increase in preventive measures, for example, by being aware of toxic elements that should not be left where children can have access.

The pediatric population in the Region of Moldova of Romania is approximately 800,000 patients, and annual admissions to “Saint Mary” Emergency Hospital are approximately 150,000/year [29]. Numerous accidental poisonings have been described at this hospital, with medicated or non-medicated chemical compounds, usually used in the household, carbon monoxide, or insecticides [29].

The results of this study underline clinical, biochemical, and esterase activity in organophosphorus pesticide exposure and its impact on children’s health. Evaluation of the pesticide exposure biomarkers in correlation with the clinical assessment is essential for diagnostic and successful treatment in case of intoxication and to improve strategies for the prevention and management of pediatric poisoning. In the light of recent research, there is a need to identify environmental and toxicological aspects to inform future best practice management for pesticide use. It can remain effective while ensuring minimal environmental contamination and with no impact on human health. This work aimed to clarify any health-related concerns about pesticide exposure and consumption in children in light of the controversies that have been published in the literature in the last few years regarding the adverse impact on humans.

We involved 49 cases of pesticide intoxication in this study. The incidence of intoxication seems to be lower in other hospitals; for example, the pediatric emergency department of Chang Gung Hospital in Taiwan, from 2011 to 2015, reported only two cases of pesticide poisoning [27].

Diazinon, an organophosphorus pesticide, was involved in 80% of the investigated poisoning cases. The literature results showed that infants and preschoolers accounted for the highest number of poisonings, which is generally in line with specialized studies [24]. Children in these age groups are generally curious but cannot judge dangers. At the same time, the literature claims that the number of cases of poisoning in adolescent children has increased, and poisoning in adolescent children has been significantly associated with suicide [28]. It is also mentioned that child poisoning rates differ between regions. In rural areas, this type of incident is more common. In addition, poisoning rates due to attempted suicide of children are higher in low-income families or with a low level of the father’s education [28]. Among the most common methods for death by suicide in children is the ingestion of poisons, including overdose pesticide ingestion, close to use of firearms, and suffocation [26,30]. Organophosphorus compounds exert their toxicological mechanism through non-reversible inhibition of the acetylcholinesterase enzyme (AChE). Among these compounds, phorate was ingested in a case of intentional suicide in a 24-year-old Bengali male [31]. Some studies also highlighted the effect of the combined toxicity potential of heavy metals and pesticides on health [32]. In our study, no associations of pesticides with other compounds were noted.

In our study, children aged 1–5 years had low values for RBC, HCT, and HGB, compared to the 6–11 and 12–17 year groups. Literature studies have shown a decrease in RBC, HGB, and HCT in chronic exposure to organophosphorus pesticides [33].

Regarding the number of red blood cells (RBCs), it is observed that they are in the biological reference range (3.8–5.05 × 10^6^/µL), but the low value for MCV (mean = 72.37) would suggest a decrease in hemoglobin synthesis. Thus, microcytic hypochromic anemia could result from inhibiting red blood cell synthesis due to an enzyme deficiency (D-ALA, Delta-aminolevulinic acid dehydratase) caused by exposure to organophosphorus pesticides [34].

Leukocytosis, present in the group of children aged 1–5 years (mean = 16.83) could explain an immune defense mechanism against acute exposure to organophosphorus pesticides [35].

The induction or inhibition of specific enzymes by organophosphorus pesticides results in changes in biochemical parameters. Thus, the present study aims to evaluate the influence of exposure to organophosphorus pesticides and their toxicity on liver and kidney function but also the impairment of glycemic status for each subject. Cholinesterase (ChE) is reported to be a specific biomarker of exposure to organophosphorus pesticides and was evaluated for each age group considered. Depending on enzyme activity, organophosphate poisoning based on plasma pseudo-cholinesterase activity (% of normal) can be mild (20–50%), moderate (10–20%), or severe (<10%) [28].

In our study, low values of serum cholinesterase were registered for children aged 1–5 years (1449.42 IU/L), and for the group of 12–17 years (1211 IU/L), values were much lower than the biological reference range. Decreased serum ChE was observed in all cases of acute organophosphorus pesticide poisoning, mainly due to the suppression of carboxylic ester hydrolases. Organophosphates deactivate AChE by phosphorylating the hydroxyl serine group present in the active site of acetylcholinesterase [11,36]. With the inactivation of acetylcholinesterase, acetylcholine increases in the nervous system causing overstimulation of muscarinic and nicotinic receptors. The clinical manifestations produced occur due to the stimulation of the autonomic and central nervous system and the nicotinic receptors on the skeletal muscle [37,38].

We found high values of serum creatinine in the 1–5 years age group, with values much higher than the biological reference range. Other authors showed that pesticide exposure causes various hematological parameters disorders and renal dysfunction in humans [39]. Literature studies show the metabolic pathways of organophosphorus pesticides. The absorption of organophosphates is easy by inhalation, and ingestion and also dermally, they accumulate in adipose tissue, liver, and kidneys, and are metabolized by cytochrome P450, and are then excreted in the urine [40,41,42].

Thrombocytopenia in the 1–5 years age group (minimum values of 37.1/mm^3^) and serum cholinesterase levels were correlated. As mentioned in other studies, the hematotoxicity of organophosphorus pesticides can reduce the synthesis of red blood cells and platelets [43].

The most common biochemical markers of liver damage are AST and ALT. Some studies evaluated the liver function of agricultural workers. They showed a direct correlation between exposure to organophosphorus pesticides and serum transaminase enzymes (ALT, AST) [44].

In many cases of acute intoxication with organophosphorus pesticides, diazinon was the offending substance. In the present study, 80% of the investigated cases reported acute exposure to diazinon (Table 1). Acute exposure to diazinon can cause hepatocyte hypertrophy and enlargement, cytoplasmic vacuolation, and macrovascular steatosis [44].

The results in the case of serum glucose are much higher than the biological reference range and were increased significantly in the case of young children, of the age range 1–5 years, with average concentrations of 157.7 mg/dL, even determined values of 513 mg/dL (Table 2). Some studies have shown a direct correlation between glycemic status, the severity of intoxication in children, serum cholesterol levels, and the total dose of atropine required for complete recovery [45]. Many theories have associated very high serum glucose concentrations following acute intoxication with organophosphorus pesticides as a sum of several mechanisms. It can also be a predictor of morbidity and mortality in cases of severe intoxication [45].

Acute organophosphorus pesticide exposure in children can lead to multisystemic inflammation in children. As seen in Table 1, most pediatric patients were neurologically affected, with toxic neuropathy, depressive disorder, and coma. More and more studies have reported neurobehavioral effects associated with acute or chronic exposure to organophosphorus pesticides in children [8]. They show only a partial quantification of the effects on the cognitive development of children exposed to organophosphorus pesticides because, depending on the mode of exposure, some neurological deficits occur over time. Regarding the evaluation of serum ChE concentrations depending on the hospitalization diagnosis, it is observed that the concentration profile in patients diagnosed with coma is different compared to other pathologies (Figure 2), and was especially characteristic of the 1–5 years age groups and the 12–17 years age groups.

The real reasons behind self-intoxication are challenging to assess among pre-adolescents and adolescents, as they are believed to be doing it for demonstration purposes. Family conflicts, lack of affection, dysfunctional family, growing up without parents, or even a rejecting–neglecting parenting style is a significant cause of triggering suicidal thoughts and attempts in adolescence [46]. Studies have shown that exposure to organophosphorus pesticides produces a different behavioral attitude due to both inhibition of serum ChE and changes in serotonin in the central nervous system, which could induce the onset of depression and subsequent suicide attempts [47,48,49]. In many cases of human poisoning with organophosphorus pesticides, the general acute symptoms are those published by the World Health Organization and are accompanied by central effects such as confusion, dizziness, headaches, and eventually coma and respiratory failure [49,50].

Regarding the possible doses that cause the poisoning, Sheldon et al. mentioned that an estimated dose of 0.02 mg/kg/day of diazinon exposure is the possible reason for persistent hypertonicity of the extremities, in a 12-week-old girl. We estimated a greater concentration of pesticides in our cases because the principal poison route is pesticide product ingestion [51].

Organophosphorus poisoning management includes immediate assessment of airway disturbances, breathing, circulation, and skin/gastric decontamination. Moreover, further steps based on risk assessment during continuous monitoring (decontaminate and limit absorption, enhance elimination, supportive therapy, close observation for 24–48 h after therapy stopped, and complete recovery) are necessary [28]. Antidotes administered are muscarinic antagonists such as atropine and oximes such as pralidoxime [15,29]. In our study, in some cases, atropine was necessary for the high intake. The administration of the antidote is carried out in the shortest possible time, sometimes the data available in the medical records suggest the administration of the antidote has already been executed during the transport to the hospital with the emergency medical team. Treatment also included decontamination, supportive care, and recommendations to be closely monitored to avoid a new incident. One of our cases died, and for all other cases, the clinical and biological evolution was favorable, with normal cholinesterase values at discharge from the hospital, with no readmission, as described in similar cases by Nistor N. et al. [29].

The pieces of information from this study can also help physicians to improve the management of pediatric poisoning cases and allows public health authorities to increase the focus of poisoning prevention efforts. Therefore, this study draws attention to the fact that increasing awareness of the danger is also necessary for parents, to handle and keep such objects inaccessible to children.

Future research directions may also evaluate the exposure and consumption degree of pesticides underlying the safe use of glyphosate in our country. Such a study would have a massive impact on many levels, especially in toxicology and public health, due to the evaluation of the possible degree of pesticide exposure and the risk associated with the population. The incidence and severity of poisoning with organophosphorus pesticides can decrease significantly through appropriate strategies. Studies on vulnerable populations such as pregnant women or professionally exposed people to harmful compounds such as pesticides are very important.

## 5. Conclusions

In conclusion, this study highlighted the toxicological effects of acute exposure to organophosphorus pesticides in pediatric patients. An extensive paraclinical evaluation was carried out regarding the results of the pediatric patients’ biological parameters. Liver and kidney damage and changes in the glycemic status were observed according to the age of each exposed subject. Cholinesterase was a specific biomarker in acute exposure to organophosphorus pesticides. The symptomatology included toxic encephalopathy, depressive disorders, and even coma among teenagers.

This study strongly supports other evidence on the toxicological potential of pesticides used in agricultural activities in the home environment that exposes young children to toxins that can pose severe risks to their health. Therefore, there is a great need for sustained efforts and increased awareness to prevent exposure to organophosphorus pesticide poisoning, especially among children aged 1–12 years. At this age, children lack judgment and the ability to assess danger, and the effects of exposure to these compounds can sometimes be fatal.

## Figures and Tables

**Table 1:** Clinical and demographic characteristics of the investigated group.

Characteristics	Details
number of cases	48
environment (urban/rural)	4/44
age groups	1–5 years (*n* = 31) 6–11 years (*n* = 6) 12–17 years (*n* = 11)
diseases due to acute intoxication with organophosphorus pesticides	toxic encephalopathy (*n* = 21), coma (*n* = 8), depressive disorder (*n* = 5), gastrointestinal disorders (*n* = 13), respiratory failure (*n* = 6)
type of intoxication (voluntary/accidental)	9/39
entry route (ingestion/dermal absorption)	46/2
place of exposure	at home
name of the involved pesticide (trade name)	Diazinon (80% of cases), others like Lindavet, Calypso, and Byemite (20% of cases)

**Table 2:** Descriptive statistics for hematological parameters.

Parameter	Children 1–5 Years (*n* = 31)				Children 6–11 Years (*n* = 6)				Adolescents 12–17 Years (*n* = 11)			
	Mean	Stdev	Median	Range	Mean	Stdev	Median	Range	Mean	Stdev	Median	Range
WBC (×10^3^/µL)	16.83	9.47	13.1	4.68–42.69	11.89	4.75	11.76	5.9–18.59	14.18	5.22	13.27	7.65–25.44
NEU (%)	66.56	20.66	71.7	8.76–90.7	70.93	11.99	72.8	55.7–88.5	75.48	17.08	80.9	29.7–93.9
LY (%)	23.06	16.96	19.85	4.28–66.6	20.6	8.8	21.35	7.5–31.2	18.16	16.26	13.5	3–64.7
MO (%)	7.23	8.14	5	1.2–48.8	7.23	3.12	6.8	3.3–11.5	5.35	1.77	5	2.9–8.7
EOS (%)	2.48	6.71	0.9	0–38.2	1.02	0.96	0.85	0–2.9	0.9	0.82	0.6	0–2.5
BAS (%)	0.51	1.66	0.2	0–9.4	0.22	0.15	0.15	0.1–0.5	0.21	0.09	0.2	0–0.3
RBC (×10^6^/µL)	4.47	0.89	4.52	0.26–5.74	4.41	0.4	4.32	4.01–4.94	4.45	0.5	4.65	3.26–4.97
HGB (g/dL)	10.92	1.89	11.3	3–13	12.18	0.74	12.15	11.2–13.3	12.66	1.21	12.8	10.5–14.8
HCT (%)	33.11	6.59	34	0.60–40.4	36.63	2.53	35.9	33.7–41.3	38.6	4.77	38.1	30.5–47.8
MCV/fL	72.37	14.37	74.3	4.59–88.2	83.33	3.08	83.85	78.9–86.9	86.8	5.21	86.1	79.1–96.1
MCH/pg	24.06	3.76	24.8	12.9–28.8	27.72	1.14	27.85	25.8–29.4	28.58	1.82	28.1	26.6–33.4
MCHC (g/dL)	32.74	1.95	32.9	27–36.4	33.3	1.19	32.75	32.2–35	32.95	1.73	32.4	31–36.4
RDW-Cv/fL	16.43	11.07	14.2	10.12–75.8	12.62	0.65	12.35	11.8–13.6	12.77	0.66	12.9	11.8–13.9
RDW-SD/fL	39.87	4.47	40.8	28.1–49.2	39.5	3.48	39.25	34.5–45.1	42.65	4.1	44	35.7–51.1
PLT (×10^3^/µL)	364.45	142.64	354	37.1–698	276.67	104.39	246	185–485	290	106.59	288	119–522
MPV/fL	8.4	1.34	8	6.4–12.3	9.5	1.57	9.8	7–11.8	9.25	1.31	9.4	6.8–11.1
PCT/fL	1.38	5.88	0.29	0.164–33.6	0.25	0.06	0.25	0.15–0.34	0.26	0.08	0.25	0.11–0.39
p-LCR (%)	30.36	61.66	17.75	8.4–361	26.82	10.86	22.95	12.1–43.7	29.45	8.79	31.3	12.8–42.6
PDW/fL	13.51	2.6	15	8.8–17.5	13.75	2.48	14.95	10.3–16.3	15.34	1.59	15.9	12.2–17.6

fL = femtoliters.

**Table 3:** Profile of the biochemical parameters at admission to the hospital.

Parameter	Children 1–5 Years (*n* = 31)				Children 6–11 Years (*n* = 6)				Adolescents 12–17 Years (*n* = 11)			
	Mean	Stdev	Median	Range	Mean	Stdev	Median	Range	Mean	Stdev	Median	Range
AST	66.23	161.69	29	18–942	30	8.7	28.5	19–44	26.55	12.27	26	12–51
ALT	46.94	123.58	19	12–717	21.67	11.97	21	8.00–45	13.27	6.85	11	5–27
ChE	1449.42	2779.47	180	41–12,017	2380.33	3058.14	786	290–8843	1211	2197.16	182	71–7036
Urea	30.47	9.04	28	17–52	24.17	6.28	24	14–32	25.18	9.19	23	8–45
Creatinine	0.58	0.16	0.58	0.26–1.02	0.68	0.17	0.64	0.45–0.997	0.65	0.1	0.6	0.56–0.92
Glucose	157.77	95.37	112	64–513	156.33	41.61	150.5	110–241	127.45	51.81	104	77–238

**Table 4:** Statistical correlation between biological parameters and ChE.

Clinical Parameters	r	*p*
ChE × ALT	0.47	0.001
ChE × AST	0.43	0.001
ChE × PLT	0.48	0.001
ChE × Creatinine	0.38	0.008
ChE × Glucose	0.39	0.009
ChE × RDW-cv	0.47	0.001

**Table 5:** Statistical data on selecting the most important variables for the clinical parameters was investigated according to age category.

Variable	Wilks’-λ	λ-Partial	F	*p*	R^2^	1-R^2^
Leucocyte	0.233	0.498	9.55	0.001	0.216	0.783
Urea	0.207	0.560	7.44	0.004	0.378	0.621
ALT	0.160	0.724	3.605	0.047	0.085	0.914
Creatinine	0.173	0.671	4.647	0.022	0.391	0.608
Lymphocyte	0.168	0.690	4.252	0.029	0.171	0.828

## Data Availability

Not applicable.

## Supplementary Materials

The following supporting information can be downloaded at: https://www.mdpi.com/article/10.3390/toxics10100582/s1, Table S1: Mann-Whitney U Test; Table S2: Discriminant Function Analysis Summary.
