# Supplementary material for: Clinical Assessment of Acute Organophosphorus Pesticide Poisoning in Pediatric Patients Admitted to the Toxicology Emergency Department
Source: Toxics. 2022 Oct 2;10(10):582. doi: 10.3390/toxics10100582 (PMC9609388; doi:10.3390/toxics10100582)
Supplement: Supplementary file 1 [file toxics-10-00582-s001.zip › toxics-1910515.pdf]

toxics MDPI

Supplementary Materials: Clinical Assessment of Acute Organophosphorus Pesticide Poisoning in Pediatric Patients Admitted to the Toxicology Emergency Department

Ioana-Cezara Caba, Violeta Ştreangă, Mona-Elisabeta Dobrin, Cristina Jităreanu, Alexandra Jităreanu, Bianca-Ştefania Profire, Maria Apotrosoaei, Alin-Viorel Focşa, Bogdan Caba and Luminiţa Agoroaei

Table S1. Mann-Whitney U Test

Variable	Rank Sum 1–5 years	Rank Sum 13–17 years	U	Z	*p*-value	Z adjusted	*p*-value	Valid N 1–5	Valid N 13–17	2*1sided exact *p*
WBC	295.0000	170.0000	104.0000	0.00000	1.000000	0.00000	1.000000	31	11	1.000000
NEU%	265.0000	200.0000	75.0000	−1.24806	0.212011	−1.24806	0.212011	31	11	0.215834
LY%	321.0000	144.0000	78.0000	1.11895	0.263163	1.11907	0.263110	31	11	0.267972
MO%	308.0000	157.0000	91.0000	0.55947	0.575839	0.55972	0.575669	31	11	0.581616
EOS%	298.5000	166.5000	100.5000	0.15063	0.880270	0.15110	0.879898	31	11	0.865570
BAS%	289.5000	175.5000	99.5000	−0.19366	0.846439	−0.20040	0.841170	31	11	0.832396
RBC	283.5000	181.5000	93.5000	−0.45188	0.651354	−0.45218	0.651136	31	11	0.641194
HGB	226.0000	239.0000	36.0000	−2.92648	0.003428	−2.92876	0.003403	31	11	0.002374
HCT	232.0000	233.0000	42.0000	−2.66826	0.007625	−2.66856	0.007618	31	11	0.006124
MCV	207.5000	257.5000	17.5000	−3.72265	0.000197	−3.72307	0.000197	31	11	0.000043
MCH	206.0000	259.0000	16.0000	−3.78721	0.000152	−3.78890	0.000151	31	11	0.000033
MCHC	290.0000	175.0000	100.0000	−0.17215	0.863323	−0.17222	0.863263	31	11	0.865570
RDW-Cv	350.5000	114.5000	48.5000	2.38852	0.016917	2.39065	0.016819	31	11	0.014154
RDW-SD	243.5000	221.5000	53.5000	−2.17334	0.029755	−2.17455	0.029664	31	11	0.026435
PLT	322.5000	142.5000	76.5000	1.18350	0.236611	1.18377	0.236506	31	11	0.232364
MPV	264.0000	201.0000	74.0000	−1.29109	0.196672	−1.29253	0.196174	31	11	0.200140
PCT	321.0000	144.0000	78.0000	1.11895	0.263163	1.11895	0.263163	31	11	0.267972
P-LCR	240.0000	225.5000	50.0000	−2.32397	0.020128	−2.32397	0.020128	31	11	0.018310
PDW	245.5000	219.5000	55.5000	−2.08727	0.036865	−2.08820	0.036781	31	11	0.033364
PDW	265.5000	199.5000	75.500	−1.22654	0.219997	−1.22818	0.219380	31	11	0.215834

Table S2. Discriminant Function Analysis Summary

Variable	**Wilks'**	**Partial**	**F-remove**	***p*-value**	**Toler.**	**1-Toler.**
MCH	0.1324	0.8778	1.3222	0.2900	0.0996	0.9004
PDW	0.1187	0.9794	0.1993	0.8210	0.2487	0.7513
MPV	0.1209	0.9616	0.3789	0.6897	0.2512	0.7488
WBC	0.2332	0.4986	9.5543	0.0013	0.2164	0.7836
Urea	0.2073	0.5608	7.4402	0.0041	0.3783	0.6217
ALT	0.1604	0.7249	3.6051	0.0471	0.0855	0.9145
PLT	0.1464	0.7944	2.4591	0.1123	0.2016	0.7984
RDW-SD	0.1530	0.7597	3.0046	0.0735	0.1488	0.8512
MCV	0.1214	0.9577	0.4198	0.6631	0.0553	0.9447
Creatinine	0.1731	0.6715	4.6476	0.0227	0.3915	0.6085
LY%	0.1683	0.6908	4.2530	0.0298	0.1711	0.8289
PDW	0.1400	0.8304	1.9406	0.1710	0.4021	0.5979
NEU%	0.1404	0.8280	1.9728	0.1665	0.1502	0.8498
AST	0.1327	0.8759	1.3456	0.2841	0.1054	0.8946
